# Emerging Glass Industry Patterns in Late Antiquity Balkans and Beyond: New Analytical Findings on Foy 3.2 and Foy 2.1 Glass Types

**DOI:** 10.3390/ma15031086

**Published:** 2022-01-30

**Authors:** Roman V. Balvanović, Žiga Šmit

**Affiliations:** 1 Laboratory of Physics, Vinča Institute of Nuclear Sciences, National Institute of the Republic of Serbia, University of Belgrade, P.O. Box 522, 11001 Belgrade, Serbia; 2 Faculty of Mathematics and Physics, Jožef Stefan Institute, University of Ljubljana, 1000 Ljubljana, Slovenia; ziga.smit@fmf.uni-lj.si

**Keywords:** late antiquity, Foy 3.2, Foy 2.1, Fe-rich, color-branding, Balkans

## Abstract

Resolving issues posed by our paper describing the late antiquity glass from Jelica (Serbia), we performed a thorough analysis of similar glass, systematically collected from the literature. The analysis showed that Foy 3.2 type evolved gradually from a composition similar to the Roman antimony-decolorized glass to a composition approaching Foy 2.1, lasting longer (second−seventh century AD) and spreading wider than originally described, including large parts of the Balkans, France interior, Germany, and Britain. The center of its distribution seems to be the Balkans and Italy. During the sixth century, Foy 3.2 glasses in the Balkans showed a significant increase of average MgO concentration compared to the earlier period and Foy 3.2 glasses outside the Balkans, implying different sand quarries and perhaps different trade routes for its imports. Recycling criteria for Foy 3.2 glass has been established. Similarly, 125 high-iron Foy 2.1 glasses are selected from the literature. They cluster within two groups regarding iron concentrations, which we term high iron (HI) and very high iron (VHI) Foy 2.1. In addition, there is a low lime subgroup of the VHI group, termed VHILL. The paper offers two possible explanations for the elevated iron, color branding, and different silica sources. High-iron glasses seem relatively evenly spread across the entire Mediterranean and its interior, representing, on average, around a quarter of the local Foy 2.1 assemblages. The percentages of high-iron samples are almost double in manufactured glass compared to raw glass, suggesting that the addition of iron was happening in the secondary workshops, i.e., for color branding. Among the manufactured glass, the proportions were higher in glassware than in windowpane glass. To capture the changing sand exploitation conditions, we propose the term “generic composition/type” or “(geochemical) class”.

## 1. Introduction

In a recent study, we described a new glass assemblage from the sixth-century from the Byzantine settlement of Jelica in Serbia [1,2]. This manganese-decolorized glass, typical of late antiquity, is characterized as Foy 3.2 and Foy 2.1 [3]. Foy 3.2 is characterized by smaller average concentrations of oxides derived from sand minerals, especially alumina (1.92%) and, to a smaller extent, lime (6.99%), indicating the use of cleaner glass-producing sands, with less heavy minerals and feldspars. Foy 2.1 is characterized by higher average concentrations of heavy-mineral related oxides like iron (1.35%), titanium (0.16%), and magnesium (1.23%), showing the use of sands with more impurities. Foy 3.2 was originally described in 17 glasses from Southern France (late fifth/early sixth century AD) and in two earlier glasses from Tunisia (late first and the second century). It has recently been recognized in several locations across the Balkans in Kosmaj [4]; Caričin Grad [5] in Serbia; Serdica, Dichin, and Odartsi in Bulgaria [6,7]; Butrint in Albania [8], and, most recently, in Slovenia [9]. Noting the similarity between Foy 3.2 and Foy 2.1 types of glass from Bulgaria, Cholakova and colleagues proposed a hypothesis that these groups are related to each other in terms of origin, but should nevertheless be considered separate primary groups. Similar observations led others to the contrary conclusion and the joint term for both groups, namely Foy 2 [10,11]. Cholakova and Rehren further discussed Foy 3.2 type in contexts from the late fourth to the early seventh century in the Western Mediterranean, Italy, and Balkans, noting a gradual increase in sand impurities over time. New glasses of Foy 3.2 type that are appearing in the literature are dated to ever-wider time-span, and show different compositions and similarities to other types such as Foy 2.1 and Roman antimony-decolorized glass. The paper focuses on the general distribution, duration, geochemical characteristics, and evolution of its composition with time, trying to interpret the reasons for this change.

Our study also found some of Jelica glasses compositionally similar to the neighboring iron-rich subgroup of the Lower Danube composition. The high-iron glass otherwise similar to Foy 2.1 was later termed high-Fe Foy 2 [10]. This glass has since been described in several assemblages [11,12,13]. A clearer picture of the characteristics and distribution patterns of the Fe-rich Foy 2.1 type is needed. The paper also examines the origin of elevated iron in these glasses.

## 2. Evolution and Characteristics of Foy 3.2-Type Glass

**Figure 1:**
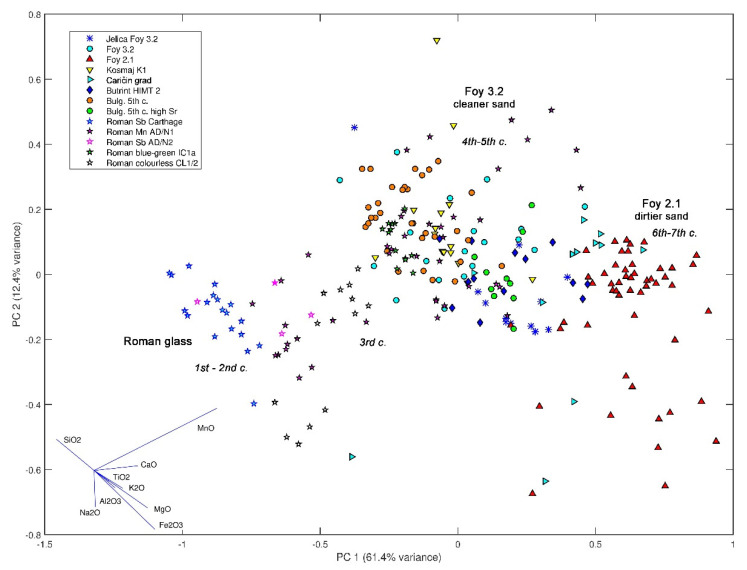
Principal component analysis of 256 glasses from 14 Roman and late antiquity natron glass groups. The diagram compares compositions of six glass groups from the Balkans, five characteristic Roman glass groups (with added manganese and with no added manganese, naturally colored and decolorized) and reference groups Foy 3.2 and 2.1. Vectors of oxides: Na_2_O, MgO, Al_2_O_3_, SiO_2_, K_2_O, CaO, TiO_2_, MnO, and Fe_2_O_3_ lower left. Data sources: (Jelica Foy 3.2—[1,2]; Foy 3.2, Foy 2.1—[3]; Kosmaj K1—[4]; Caričin grad—[5]; Bulg. 5th century—[7]; Butrint HIMT 2—[8]; Roman Sb Carthage—[10]; Roman AD/N1, AD/N2—[14]; Roman Ic1a—[15]; Roman CL1/2—[16]).

To obtain a synoptic compositional overview of the considerable diversity of the Roman and the Late Antiquity natron glass types, a large amount of data have been plotted on the principal component analysis (PCA) diagram (Figure 1). The selection of oxides (SiO_2_, Na_2_O, MgO, Al_2_O_3_, K_2_O, CaO, TiO_2_, MnO, and Fe_2_O_3_) is limited by the published data. Before PCA, power transformation ${\hat{x}}_{ij}={\tilde{x}}_{ij}-\bar{{\tilde{x}}_{i}}$, where ${\tilde{x}}_{ij}=\sqrt{x_{ij}\;}$ is performed on the data because of their heteroscedasticity (different variances across oxide values). The diagram plots five late antiquity glass groups from the Balkans classified as série 3.2 (Jelica, Kosmaj, and Caričin grad from Serbia; Butrint from Albania; and a Bulgarian fifth century composition), and compares it against several groups of typical Roman natron glass and Foy série 3.2 and série 2.1 as the referential groups. The comparison groups include Roman manganese added glass (group AD/N1), several Roman antimony added glass groups (AD/N2, Roman Sb glass from Carthage, and colorless group CL1/2), and Roman blue-green glass group Ic1a.

The PCA diagram can be interpreted chronologically. Vectors show a similar direction for an increase in oxides, reflecting both light and heavy minerals of the sand. Roughly congruent vector slopes reflect correlations between oxides reflecting light (alumina, potassium, and lime) and heavy minerals (titanium, iron, and magnesium). The change from antimony to manganese as a decolorizer, which started around the end of the third century [17], is manifested on the diagram by a shift in the direction of the manganese vector. Note that since manganese gradually replaced antimony as a decolorizer, the direction of the manganese vector on the PCA diagram roughly corresponds with the physical time for the period of the third−fifth century. Analogously, the direction of time in the period of the fifth−sixth century is roughly parallel to the vectors of oxides representing the heavy minerals of the sand.

There are three major compositional aggregations: Roman glass with no deliberately added manganese (left), Roman manganese-added glass and Foy 3.2 (middle), and Foy 2.1 glass (right). Oxide vectors, showing the directions of increase of corresponding oxides concentrations in the PCA diagram, demonstrate that the central aggregation differs from the left one mostly by the increased manganese. Analogously, the aggregation on the right side differs from the central one by a marked increase in heavy and light sand minerals.

How does Foy 3.2 differentiate from these glass groups? Compared to the “Roman glass” assemblage [18], Foy 3.2 has lower alumina and lime. Foy 3.2 has lower Al_2_O_3_ (1.92 ± 0.15% versus 2.46 ± 0.15%) and, to an extent, CaO (6.99 ± 0.74% versus 7.74 ± 0.53%) than Roman manganese-added group AD/N1, and a lower sum of light sand minerals (0.138% versus 0.158%), indicating the use of sand with less feldspars and lime (Table 1). Compared with the other major Roman glass types without added manganese, the antimony-added group AD/N2, Foy 3.2 differs by having higher lime (6.99% versus 5.14%) and alumina (1.92% versus 1.77%), as well as increased concentrations of oxides reflecting both heavy minerals (average of 2.1% against 1.4%) and light minerals (13.8% versus 11.2%), but it is also in lower zirconium (48 ppm for versus 73 ppm). This reflects different sand and lime sources, and different suits of heavy minerals. Regarding sand provenance, the TiO_2_/Al_2_O_3_ ratio discriminates oxides reflecting heavy minerals rich Egyptian sands from alumina rich Levantine sands [10]. This ratio is around 2.5% for Levantine sand and almost double and more for the Egyptian sands. While manganese added Roman glass group AD/N1 is of a Levantine origin (TiO_2_/Al_2_O_3_ ratio is 0.024), the antimony added group AD/N2 and série 3.2 originate in Egypt (0.047 and 0.049, respectively).

Foy 2.1 is differentiated from série 3.2 by a significant increase in the sum of all oxides reflecting minerals in the sand (21.6% versus 15.9%) and in the TiO_2_/Al_2_O_3_ ratio (0.062 versus 0.049). It has double the concentration of heavy sand minerals compared to Foy 3.2, and around 25% higher light minerals. Taking into account standard deviations, we propose the cutoff values for differentiating Foy 3.2 and 2.1 to be 0.03 for oxides reflecting heavy minerals in the sand, 0.16 for light minerals, and 0.19 for the sum of oxides reflecting both heavy and light minerals. There is a question regarding the alumina cutoff value between Foy 3.2 and 2.1. D. Foy reports alumina concentrations of 1.92 ± 0.15% for série 3.2, and 2.54 ± 0.15% for group 2.1 [3], allowing the cutoff might be set to, e.g., 2.3%. However, several papers have since reported Foy 3.2 type of glasses with higher alumina concentrations. The examples include alumina concentrations of up to 2.38%, 2.51%, and 2.59% [7,19,20], so higher alumina concentrations for Foy 3.2 should be allowed. What differentiates Foy 2.1 from 3.2 in such cases is the still higher heavy mineral concentrations and TiO_2_/Al_2_O_3_ ratios.

Another yet unaddressed issue regarding série 3.2 is that it includes two non-tardifs samples from the first−second century AD, far earlier than the rest of the group. While these two are indeed very similar to the 17 samples dated to late fifth−early sixth century (being somewhat lower in light sand minerals), a question arises regarding the huge time-span between them. Indeed, D. Foy noted that série 3.2 is not specific for a single period, but that sand for its manufacturing was exploited for a longer period, and that other deposits were surely exploited at the same time [3]. This opens a question regarding the time duration of Foy 3.2 type. Whether the time gap between the two non-tardifs série 3.2 samples and fifth−sixth century série 3.2 is perhaps filled with assemblages dated continuously from the early second century to the sixth century AD? This time gap has been filled up, with several authors reporting Foy 3.2 assemblages from different periods and places. These include collections from the fifth century Bulgaria [7], fourth−fifth century Italy [19], late third−sixth century Italy [21], mid fourth−fifth century Germany [20], fourth century England [17], third−fourth century AD, outlier sample YAS-265 from Carthage [10], and second−fourth century AD Kosmaj in Serbia [4]. It seems that in the case of Foy 3.2 type, there is a need to overcome the traditional definition of a compositional group being tied to a single place and time of sand exploitation, and to introduce a definition that would encompass a greater area and longer time.

**Figure 2:**
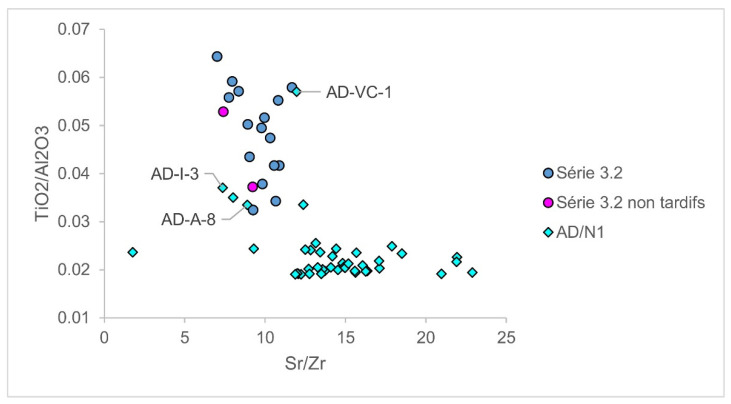
Provenance indicators. TiO_2_/Al_2_O_3_ versus Sr/Zr, indicators of sand heavy minerals for Foy série 3.2 and Adria AD/N1 show that glasses AD-A-8, AD-I-3, and AD-VC-1 from AD/N1 (of Levantine origin) more likely utilized Egyptian than Levantine sand. Data sources: [3,14].

With the mentioned criteria, we searched the literature and carefully selected a total of 246 glasses that could be attributed to Foy 3.2-type (Supplementary Material Table S1, Foy 3.2 recycling). Many glasses are attributed as Foy 3.2 for the first time, and some are reattributed. Each new attribution and reattribution were performed very carefully, using all available compositional data and testing extensively through numerous diagrams. Indeed, some of these glasses seemed to further expand the timeframe of série 3.2 and fill the mentioned time gap, like AD-A-8 and AD-I-3 from Adria, dated from the second half first to the early second century and the third century AD, respectively. Their elevated TiO_2_/Al_2_O_3_ and smaller Sr/Zr ratios fall well within série 3.2, with an Egyptian provenance of sand, and not within AD/N1 glasses, as is the case of manganese-added Roman glasses (Figure 2). In addition, elevated hafnium concentrations of AD-A-8 and AD-VC-1 (1.2 and 1.4 ppm, respectively) compared to the pertaining AD/N1 group (0.81 ± 0.18, excluding the outlier AD-A-11 with 6.6 ppm of hafnium) conform better to Roman antimony glass AD/N2 (1.13 ± 0.22 ppm), and MSG1c group from Padova (1.41 ± 0.81 ppm), classified as Foy 3.2, both with an Egyptian provenance. As shown by Barfod et al. [22], the hafnium concentration in sands decreases from the Nile delta towards the Levantine coast during the longshore transport of the Nile sediments. This indicates that AD-A-8 comes from a location that is likely closer to the Nile delta from the origin of the majority of AD/N1 glasses. In addition, this glass on the PCA diagram is close to the early Foy 3.2 glass VRR390 from Nabeul [3], dated also to the end of the first century AD, which further strengthens such an attribution. Likewise, Adria glass AD-VC-1 is dated to the second−fourth century, and AD-I-3 to the third century AD. This gives evidence to tentatively attribute these three glasses from Adria to Foy 3.2 type. Similarly, five seventh-century glasses from Crypta Balbi in Rome [23] and two seventh-century Merovingian glasses from Vicq in France [24] possibly extend the opposite end of the Foy 3.2 timespan.

**Figure 3:**
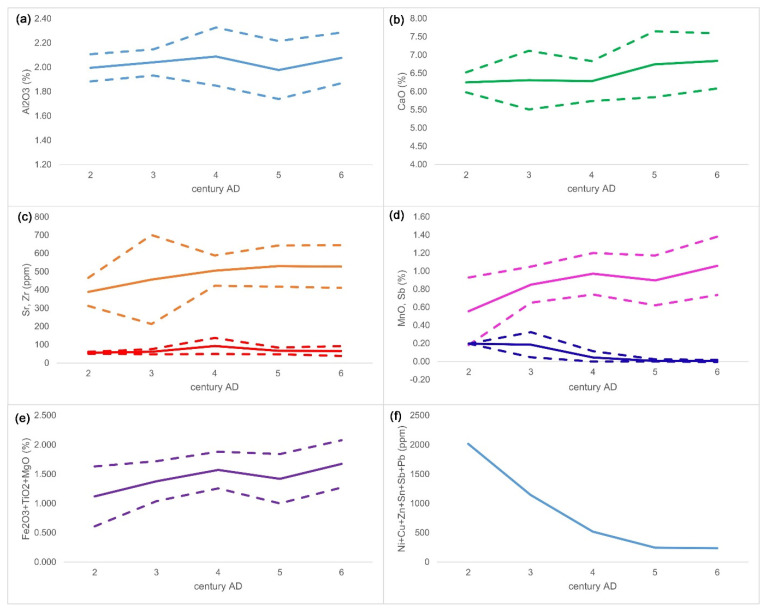
Evolution of the composition of série 3.2 in time. Selected oxides concentrations of 246 individual Foy 3.2 type glasses, belonging to 28 different glass groups across the Mediterranean and Western Europe, second−sixth century AD. Alumina (**a**), lime (**b**), strontium (orange) and zirconium (red) (**c**), manganese (magenta) and antimony (blue) (**d**), the sum of oxides reflecting heavy minerals of sand (**e**), and sum of trace elements reflecting recycling (**f**). Averages are straight lines, and standard deviations are dotted lines. Data sources: [1,2,3,5,6,7,8,9,10,12,14,19,23,24,25,26,27,28,29,30].

To try to gain some insight into the possible evolution of the composition, 246 Foy 3.2-type glasses were sorted by centuries according to their dating, and the averages and standard deviations of selected oxides and trace elements for each century were calculated and plotted (Figure 3). While some of the date attributions were indeed uncertain, many were reliable, and the observed timeframe was long enough that general trends could nevertheless be noticed with reasonable confidence. The relative stability of the mean concentrations of alumina and lime through the centuries (Figure 3a,b) indicate that sand -sources were located within a geologically similar area, poor in feldspars and lime. However, a gradual change also exists in the compositions with time. The overall tendency is a slow but steady increase of lime, strontium, zirconium (Figure 3c), iron, titanium, and magnesium, derived from heavy minerals of the sand. The sum of iron, titanium, and magnesium is 1.12% in the second century to 1.68% in the sixth century AD (Figure 3e). Antimony, derived from recycling, at first was relatively high (0.08% in group 2a of Foster and Jackson or 0.2% in VRR391), but decreased with time, as the antimony-decolorized cullet became less available, especially from the third century AD onwards (Figure 3d).

The decreasing degree of recycling is also notable, indicated by the constantly diminishing Ni + Cu + Zn + Sn + Sb + Pb sum, from over 2000 ppm in the second century to 237 ppm in the sixth century AD. A temporary exception to the trend of the increase of sand minerals is a slight change in the composition from the fourth to the fifth century AD, manifested by a decrease of alumina (2.09 to 1.98%), magnesium (0.79 to 0.65%), manganese (0.97 to 0.90%), and zirconium (93 to 67 ppm) and an increase of lime (6.29 to 6.75% on average). These changes likely reflect the use of different quarries over time [31]. It is important to stress that the time perspective or “evolution” of the composition gives quite different semantics to the meaning of a “compositional group”; while the classical compositional group reflects a single quarry and a few primary workshops in its vicinity, our approach implies a dynamic view: constant changing of sand quarries within the similar geochemical area and through a longer period. The concept tries to capture the dynamics of the process.

**Figure 4:**
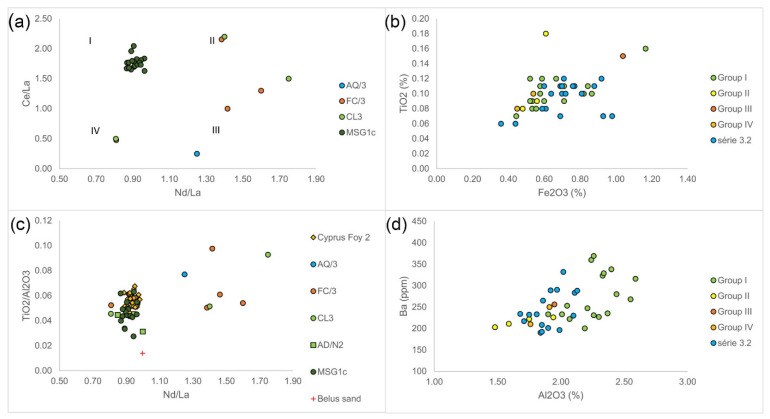
Groupings of Foy 3.2-type glasses according to trace elements. Nd/La versus Ce/La (**a**), Fe_2_O_3_ versus TiO_2_ (**b**), Nd/La versus TiO_2_/Al_2_O_3_ (**c**), and Al_2_O_3_ versus Ba (**d**). Data sources: [14,19,25,26].

Série 3.2 glasses seem to form four distinct groups depending on their respective Nd/La and Ce/La values, namely: Nd/La < 1, Ce/La > 1 (group I); Nd/La > 1, Ce/La > 1 (group II); Nd/La > 1, Ce/La < 1 (group IIII); and Nd/La < 1, Ce/La < 1 (group IV; Figure 4a). MSG1c glass forms group I while AQ/3, FC/3, and CL3 glasses are distributed over groups II, III, and IV. Groups I and II are differentiated by Al_2_O_3_ and Ba concentrations (Figure 4d), showing differences in light sand minerals, while groups III and IV are differentiated by Fe_2_O_3_ and TiO_2_ concentrations (Figure 4b), reflecting variations in heavy minerals. Groups III and IV are also differentiated by CaO, SrO, and Na_2_O concentrations, possibly showing different sources of lime and different manufacturing recipes. This suggests the variability of sands used for manufacturing these groups. Different Al_2_O_3_/SiO_2_ versus TiO_2_/Al_2_O_3_ provenance indicators of the respective groups, albeit within a wider span of values characteristic for série 3.2 glasses, seem to confirm this. It should be noted that a relatively small number of samples with measured REEs (26) limits making more definitive conclusions, but it is still sufficient to gain insight into the compositional differences among série 3.2 glasses.

Note that the AD/N2 group overlaps with the group I on Nd/La versus Ce/La diagram, further supporting the noted similarity between Roman antimony-decolorized glass and série 3.2 [10]. Note also that some groups classified as Foy 2 or Foy 2.1 share the Nd/La versus Ce/La space with group 1. This shows that group I of série 3.2 glasses has a similar origin to Foy série 2.1, while other types have not. Table 2 summarizes the Nd/La, Ce/La, and Zr/TiO2 ratios for various groups.

The different geology is manifested also in REE correlations. In Foy 3.2, La, Ce, and Pr are not correlated, whereas in Foy 2.1, all REE elements and yttrium are strongly correlated (typically R^2^ > 0.8), while for La, Ce, and Pr are to a lesser degree with Th (>0.6), Hf (0.29–0.60), Zr (≥0.3), Nb (≥0.48), and Ti (0.22–0.47), indicating the presence of heavy minerals (e.g., allanite). Contrary to CL3, high correlations between titanium, niobium, and tantalum in Foy 2 from Cyprus indicate the presence of minerals like rutile [11].

As mentioned, Foy 3.2 has two distinct Nd/La ratio spans, Nd/La < 1 (groups I and IV) and Nd/La > 1 (groups II and III). Foy 2.1 glasses share this range, averaging from 0.92 to 0.95. For comparison, Belus river sand has Nd/La = 1 [35]. Group I is similar to Foy 2.1 in this regard, but can still be differentiated by a lower Zr/TiO_2_ (around 50 versus 75). Group IV is distinguished from Foy 2.1 by a lower Nd/La and especially by a much lower Ce/La ratio (around 0.5 compared to above 1.5). Groups II and III have higher Nd/La than Foy 2.1 and HIMT glasses, above 1.2 compared to less than 1. It seems that Nd/La might be a good marker for differentiating Foy 3.2 from Foy 2.1 sand provenance, but caution should be kept because of the small number of available measurements of REEs in Foy 3.2 glasses. The ratio of Ce/La [11] for groups III and IV is on average lower than in Foy 2.1. The proxy for heavy minerals Zr/Ti, characterizing Egyptian sands, also differentiates these glasses from the HIMTa and HIMTb groups.

To determine the recycling degree of série 3.2-type glasses, we analyzed 20 raw glasses from the entire collection of 246 glasses (S1 Foy 3.2). We first eliminated glasses with values of Cu, Sb, and Pb > 100 ppm (criteria of Foy et al., 2003), then from the remaining glasses, we eliminated those with obviously elevated antimony (Sb > 10 ppm), obtaining 13 raw glasses that can be considered unrecycled. Cutoff values of the unrecycled raw glass were then determined as the mean values plus two standard deviations and were rounded up. The obtained cutoff values were 10 ppm for Sb, 40 ppm for Co, 50 ppm for Ni and Zn, 60 ppm for Pb, and 70 ppm for Cu. Above these levels, série 3.2-type glass can be considered surely recycled, and below it is modestly recycled or unrecycled.

Applying the obtained values to manufactured glasses, we obtained 54 glasses with means of 7 ppm, 26 ppm, 13 ppm, 1 ppm, and 17 ppm for nickel, copper, zinc, antimony, and lead, respectively. Eliminating the glasses with values of the recycling indicators above the mean plus two standard deviations, we obtained 35 glasses. The means plus two standard deviations of the recycling indicators can be considered as the cutoff values for pristine série 3.2-type glass. These values were 8 ppm, 37 ppm, 21 ppm, 2 ppm, and 29 ppm for nickel, copper, zinc, antimony, and lead, respectively.

Using these criteria, 14.3% of Foy 3.2 can be considered pristine, 47.1% is moderately recycled, and 38.6% surely recycled. The série 3.2 and the pristine Foy 3.2 are compositionally very similar, except for the pristine being somewhat lower in lime (6.7% versus 7%), iron (0.62% versus 0.7%), antimony (1 ppm versus 18 ppm), and lead (16 ppm versus 179 ppm). It is also mildly higher in zirconium (62 ppm versus 57 ppm) and titanium (0.1% versus 0.09%). These differences reflect a degree of recycling with other types of glasses, such as Roman Sb glass. Similar calculations were performed for pristine Levantine I glass from Cyprus, obtaining 3 ppm for cobalt and copper, and 10 ppm for zinc and lead [11]. This shows that glass of série 3.2-type was manufactured using sand somewhat richer in minerals than the Levantine, but it can nevertheless be considered quite clean.

## 3. Characteristics of Iron-Rich Foy 2.1 Glass and Source of Increased Iron

An extensive literature search yielded 125 glasses of Foy 2.1 type with elevated iron (Table 3, S2 Fe-rich Foy 2.1 correl). They are grouped in three groups regarding iron oxide concentrations: 47 high iron glasses (HI); 74 very high iron glasses (VHI); and 4 very-high iron, low lime glasses (VHILL). Apart from considerably higher average iron (2.29% versus 1.03%) iron-rich glass is also differentiated from série 2.1 by mildly higher alumina (2.75% versus 2.49%), and lower lime (7.42% versus 7.97%) and manganese (1.11% versus 1.69%). The VHILL was differentiated from VHI by lower lime (5.43% and 7.48%, respectively) and strontium (433 ppm versus 715 ppm), and lower magnesium (0.98% versus 1.32%). Taking into account two standard deviations and minimal/maximal values, we could conveniently draw the iron concentration limit between high and low iron Foy 2.1 to 1.3%, and between the HI and VHI to 2%. Similarly, we defined the lime limit between VHI and VHILL to 6%, and the upper titanium level of the entire high-iron glass to 0.2%.

**Figure 5:**
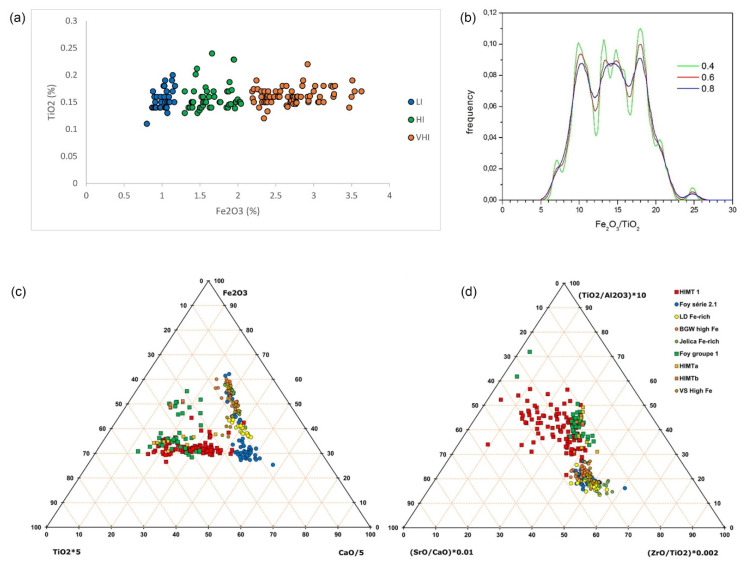
Iron and titanium distributions (up) and comparison between Fe-rich Foy 2.1 and similar late antiquity glass groups (down). Bi-plot of Fe_2_O_3_ and TiO_2_ for Fe-rich Foy 2.1 glasses, including série 2.1 (**a**). Kernel density estimation for Fe-rich glasses, for three values of parameter (h) (**b**). Comparison between Fe-rich Foy 2.1 and similar late antiquity glass groups (lower). Compositional comparison of Fe-rich Foy 2.1 glasses with similar late antiquity glass groups (**c**). Provenance indicators based on sand mineralogy (**d**). Fe-rich Foy 2.1 glasses outlined in black. Values scaled to magnify and center the distributions.

There are three glasses with TiO_2_ contents higher than the rest of the Fe-rich glasses, averaging 0.16 and 0.23%, respectively (Figure 5a), but their high lime contents (7.8% on average) disqualified them from being classified as HIMT. The frequency of iron and titanium concentrations was also estimated by the kernel-density estimate (KDE) [36]. The optimal value of the bandwidth parameter (h) was sought by trial and error. In Figure 5b, we present distributions for three different values of h, for which we estimated the most representative for the density variation. The diagram shows three peaks in Fe_2_O_3_/TiO_2_ distribution, centered at around values of 12, 14, and 18, respectively, indicating different mineral compositions. However, we need more glasses of this type to make firmer conclusions. Iron in Fe-rich glasses is not in correlation with trace elements indicative of recycling (NiO, CuO, ZnO, SnO_2_, Sb2O_3_, and PbO), nor with pollutants (SO_3_). It also does not have a negative correlation with Cl, which would indicate recycling, so its source needs to be found elsewhere.

Compared to other glass groups with elevated iron, Fe-rich Foy 2.1 is equal in iron to Foy 1 (2.29% versus 2.28%) [3]. However, its considerably lower titanium (0.16% versus 0.49%) and zirconium (114 ppm versus 216 ppm) differentiate the two. Its iron levels are intermediate between HIMT 1 (1.36%, [37]) and very high-iron HIMT groups like IIa Dichin 2.91% [38], AQ/1a 3.23% [21], FC/1a 3.69% [26], and CL1a, 3.38% [19]. HIMT glasses are analogously differentiated by iron levels (HIMTa and HIMTB, [32], also reported by [19,21]). While Foy 2.1 HI and HIMTa are on average comparable in iron (1.64% versus 1.81%), VHI is lower than HIMTb (2.69% versus 3.55%). In addition, in HI and VHI groups, iron and titanium are not correlated, contrary to HIMTa and HIMTb, indicating different mixtures of heavy minerals in the sand (like zircon, pyroxenes, and amphiboles).

Correlations of strontium with manganese (0.54) and of manganese with SrO/CaO ratio (0.6) in the HI group indicate that a part of its strontium comes from manganese ore. The SrO/CaO trendline extrapolates backwards at around 70, above the values characteristic of low-manganese Levantine glass (45–55). However, a part of Sr in Egyptian sands might also come from other Sr-bearing minerals or from the diagenetic alteration of aragonite into calcite [11]. The ratio of SrO/CaO in VHI is 88, intermediate between Egypt (around 70), and HI (98). VHILL is lower in Ba and Sr than VHI. As they have different Sr/Ca ratios (80 versus 96), this difference might come from sand with different amounts of Ba-bearing feldspar and Sr bearing minerals like witherite and strontianite.

The correlation of zirconium with titanium of the HI group is similar to the Egyptian glass (0.63 versus 0.56, [39]), but its Zr/Ti ratio is smaller (54 in HI and 59 in VHI versus 84 in Egypt), indicating different heavy minerals suites. Positive correlation Zr-Sr (0.58) is not congruent with regional geology, exhibiting zero or negative values. It is comparable to the Bulgarian Fe-rich (0.42), Cypriot Foy 2 (0.45), and Foy 3.2 (0.53). This indicates possibly different sand sources for these groups.

Figure 5c depicts some of these considerations. The iron-rich Foy 2.1 glasses are separated from HIMT groups by lower titanium and higher lime. Considering provenance, the TiO_2_/Al_2_O_3_ ratio, differentiating heavy mineral-rich Egyptian sands from feldspar-rich Levantine sands [10], is compared with ratios of zirconium to titanium, characterizing regional sands, and of strontium to calcium, differentiating coastal from inland sands. Provenance regions for HIMT and Fe-rich Foy 2.1 are also clearly separated (Figure 5, right). This is further supported by their different Ce/La ratios of 1.51 and 1.2, respectively.

As mentioned by Ceglia et al. [32], the Ce/La ratio can be used for sand characterization. In this sense, it is noteworthy that in Fe-rich Foy 2.1 glasses, the Ce/La ratio decreases with the increase of iron, manifesting a strong negative correlation of 0.8 in Byzantine glass weights (BGW) and 0.85 in Cypriot Foy 2. The negative correlation of Ce/La with iron is also seen in HIMTa and HIMTb, although less pronounced (0.7) and with higher Ce/La ratios for the same concentration of iron, indicating different sand sources for iron-rich Foy 2.1 and HIMT glasses. Another possible provenance indicator is Ce/Gd, the ratio between the most abundant light and heavy REE element. Light and heavy REEs have a different hydrothermal mobility and reflect different hydrochemical and geochemical processes [40,41]. For VHI/VHILL, Ce/Gd is around 6.9, lower than for Levantine, Foy 2, and Egypt1 (all above 9), and comparable to HIMTb (6.5). This ratio is also negatively correlated with iron. In addition, Fe-rich Foy 2.1 glasses are differentiated from HIMTa and HIMTb by lower zirconium (around 100 ppm compared to above 200 ppm) and hafnium (2.1–2.4 ppm compared to 5.2 and 5.7 ppm), while the correlation of hafnium with zirconium varies more (0.66–0.98 compared to 0.98 and 0.96). The Byzantine glass weights (both Foy 2 and Fe-rich Foy 2) have strong correlations between REEs, while Foy 2 from Cyprus is mainly between the LREE. In addition, Ce and Gd are less correlated with other REEs in Fe-rich than in Foy 2 Byzantine glass, indicating different heavy minerals suits.

Regarding the origin of high iron, several assumptions can be made. Emphasized recycling signs allow for the hypothesis that this glass is perhaps manufactured by mixing low-iron Foy 2.1 with some contemporary available high-iron cullet. For example, mixing it with 60% glass similar to Dichin 2b HIMT or with 70% glass similar to Cyprus HIMTb yields adequate values of iron, alumina, and strontium, but higher titanium and zirconium and lower lime than expected. The composition of the hypothetical cullet needed to obtain Fe-rich Foy 2.1 composition, in the proportion of 70% of cullet and 30% of low-iron Foy 2.1 (similar percentages are reported by Silvestri 2008), comprises 3.6% iron, 0.17% titanium, 3% alumina, 7.3% lime, 128 ppm zirconium, and 727 ppm strontium. We are not aware of such contemporary glass. Other authors also exclude mixing with HIMT based on REE patterns [6].

Another hypothesis is the exploitation of specific, iron-rich sand quarries from the geologically related area, as evidenced by the similarity of their respective REE patterns (e.g., between Foy 2 and Fe-rich Foy 2 glasses from Cyprus and LI and HI glasses from Visighotic Spain). The case of different iron concentrations in Hambach glass factories [42,43], ranging from 1.4% to 1.8% to 2.2% on average, further makes this hypothesis plausible.

Technological interpretations for increased iron should also be considered, like contamination from oxidized scales from iron blow-pipes that forms during glassmaking [44], but the difference in trace elements ratios does not support this hypothesis.

Another candidate is the color-branding hypothesis [45]. This states that the HIMT glass was deliberately tinted by manganese ore to color-brand its superior working characteristics. Two different manganese ores were used according to the hypothesis, with different iron to manganese ratios, yielding two different types of glass, HIMTa and HIMTb, that are differentiated by Fe_2_O_3_/Al_2_O_3_ and Fe_2_O_3_/TiO_2_ and Ce/La ratios [11,32].

**Figure 6:**
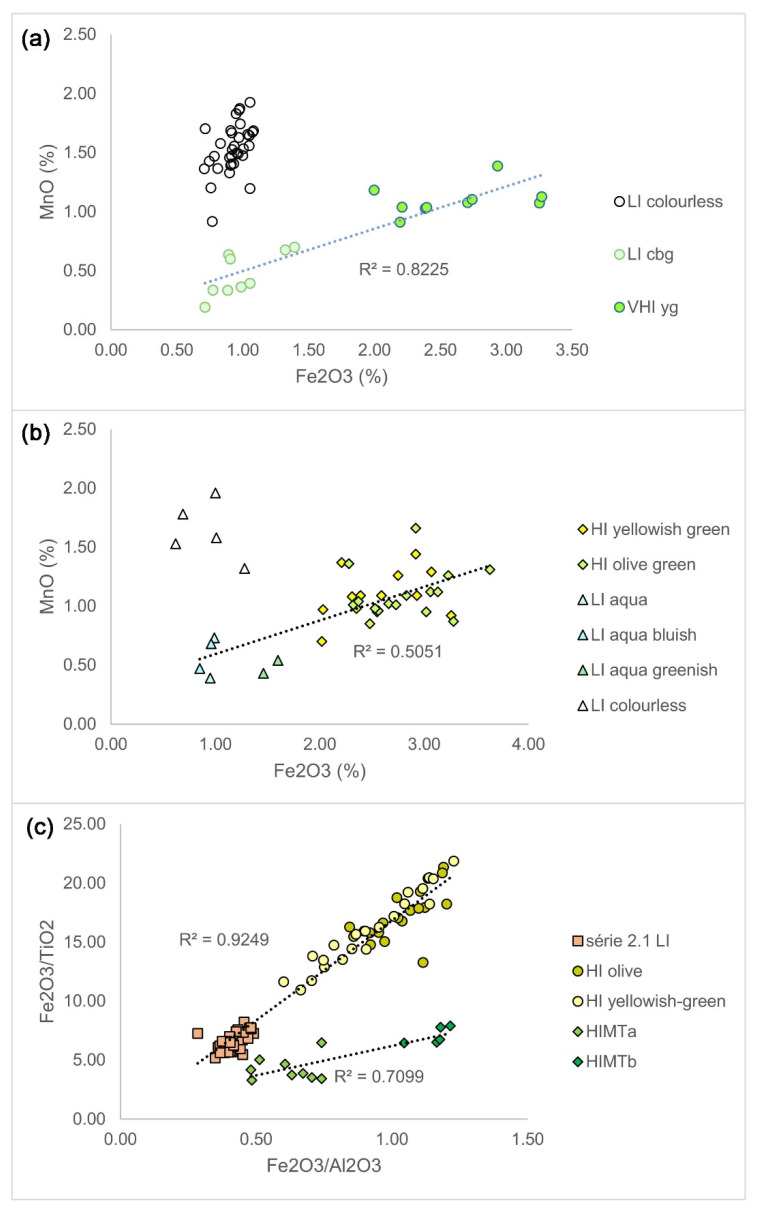
Color-branding hypothesis. Correlation between Fe_2_O_3_ and MnO for Foy 2.1 glasses from Visighotic Spain (VS) (**a**), Byzantine glass weights (BGW) (**b**); Fe_2_O_3_/Al_2_O_3_ versus Fe_2_O_3_/TiO_2_ diagram of low iron Foy 2.1 and all high iron Foy 2.1-type of glasses, grouped according to color (**c**). HIMTa and HIMTb glasses from Cyprus are given for comparison. Data sources: [3,11,12,13].

Figure 6 shows a diagram of Fe_2_O_3_ versus MnO for Foy 2.1 assemblages from Visighotic Spain (upper) and Byzantine glass weights (middle). The low iron glasses form two distinct groups: lightly colored (mostly colorless, bluish and greenish) low iron glasses (LI cbg) and LI colorless glasses. The colorless glasses are decolored with manganese (MnO/Fe_2_O_3_ = 1.67). The very high iron glasses are darker colored, green and yellow (VHI yg). Lightly colored LI glasses lie on the same correlation line with darker colored VHI glasses (0.82). There is no such correlation for HI glasses. In the BGW collection, lightly colored LI glasses (different shades of aqua) fall on the manganese−iron correlation line with high iron olive and yellowish glass from the same collection (R^2^ = 0.51). Considering only olive-green HI glasses, the correlation is more pronounced (0.59). Colorless low iron glasses from BGW assemblage are decolored with manganese (MnO/Fe_2_O_3_ = 1.7). Some low iron glasses in BGW assemblages are of an amber color (not shown for clarity). Their average manganese to iron ratio is similar to the decolorized glasses (1.65), but might derive their color from ferri-sulphide. The pronounced manganese−iron correlations between pale low iron glasses and darker high iron glasses in these two collections supports the color branding hypothesis.

A strong correlation between Fe_2_O_3_/Al_2_O_3_ and Fe_2_O_3_/TiO_2_ ratios for HIMTa and HIMTb glasses from Cyprus, noticed by Ceglia and colleagues, also exists for the set of low iron and high iron Foy 2.1 glasses. (Figure 6, lower). The diagram shows this correlation between the low iron série 2.1 glasses of all colors and the most common Foy 2.1 high iron glasses, olive and yellowish. Different correlation lines of Foy 2.1 and HIMT glasses suggest that different manganese ore was used for color branding.

**Figure 7:**
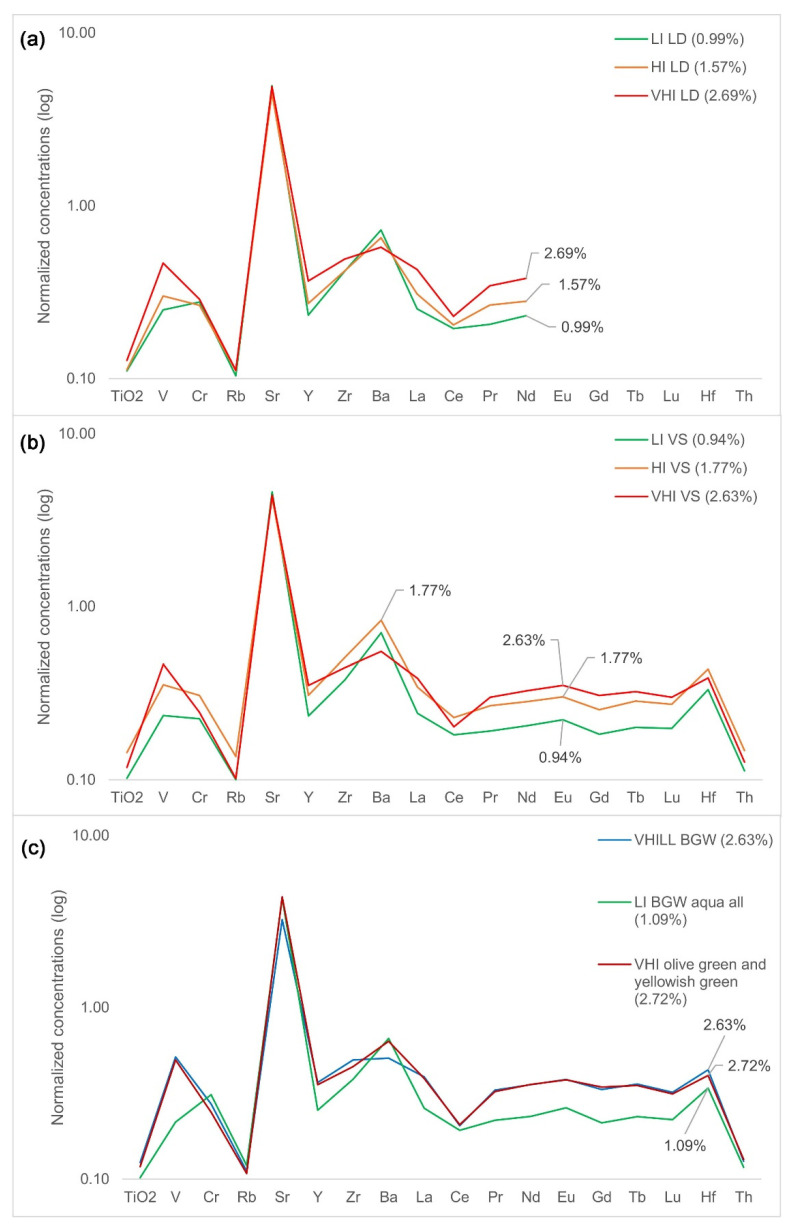
Trace element patterns of Fe-rich Foy 2.1 glasses, grouped by iron concentrations. Values normalized to the upper continental crust [46]. Groups from Lower Danube (**a**), Visighotic Spain (**b**), and Byzantine glass weights (**c**). Note that for the Lower Danube REE dataset, only La−Nd measurements are reported. Data sources: [6,12,13].

Figure 7 shows normalized REE patterns for glasses from Lower Danube (LD), Visigothic Spain (VS), and Byzantine glass weights (BGW), grouped by iron concentrations. With an increase in iron, the concentrations of REEs in all three compared datasets increased. An exception to this is HI glass compared to VHI glass from the Visighotic collection, where cerium and hafnium decrease with an increase of iron concentrations in high iron glasses, indicating a different type of mineral iron. Another discrepancy is the decrease of barium with an increase of iron in the same assemblage and the Lower Danube collection. The observed tendency is related to strontium. Correlations of barium with strontium are around 0.8 for HI glass from Lower Danube and Visigothic Spain, respectively.

The REE patterns of low iron and high iron Foy 2.1 glasses from Visighotic Spain (Figure 7, middle) are very similar, which implies that differences in their heights are primarily related to iron concentrations (0.94% versus 1.77%). This implies a similar silica source but with higher concentrations of iron- bearing minerals, in other words, iron-based coloration. However, the REE pattern for VHI glass from the same collection is different from the HI REE patterns, which suggests different mineral compositions or the deliberate addition of some minerals to the glass-making mixture. Adding iron-bearing manganese ore, used for color branding, might account for this difference in REE concentrations. This would suggest that HI glasses from Visighotic Spain are colored naturally while VHI glasses are color branded. Indeed, almost all VHI glasses from this set are green-yellow, which is not so for the HI glasses from the same collection. Likewise, VHI Byzantine glass weights also seem color branded. Another sign of this is the average correlation of 0.47 between manganese and REEs for the set of olive and yellowish-green VHI and aqua LI BGW, quite similar to the correlation between manganese and iron for the same set (0.51). The relative decrease of cerium in these glasses with a higher iron can be accounted for by the fact that iron is not correlated with cerium, while it is well correlated with other REEs for the same collection (0.74).

This leaves us with two plausible hypotheses for the origin of high iron in Foy 2.1 glass, namely: variability of sand source and the color-branding. Both might even be correct, depending on the particular glass group. The candidates for the former are HI and LD VHI groups, with LD VHI having strong correlations of LREEs with iron and magnesium, and none with manganese, indicating that REEs are related to the heavy mineral fractions of the sand. The candidate for the latter is Fe-rich Byzantine glass weights, where iron and magnesium are not correlated with REEs. They are almost all olive green or yellowish-green (contrary to Foy-2 glass weights that are of many different colors), further supporting the color-branding hypothesis.

## 4. Discussion

### 4.1. Changes in Distribution of Glass Types from Fourth to Sixth Century AD in the Balkans

**Figure 8:**
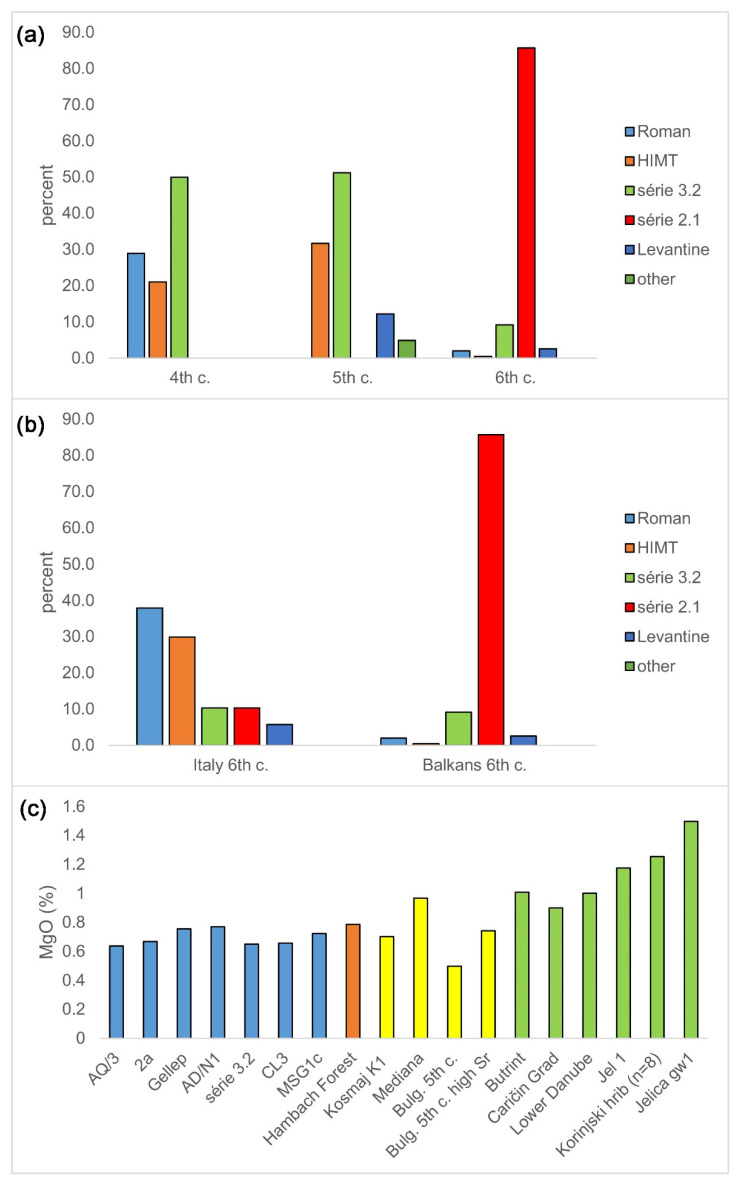
Relative frequencies of glass types by time and region and MgO concentrations by location. Relative frequencies of glass types from fourth century (38 glasses), fifth century (82 glasses), and sixth century contexts (196 glasses) from central and eastern Balkans (**a**). Relative frequencies of compositional types among glasses from sixth century contexts from the central and eastern Balkans (196 glasses from three locations) and northern Italy (87 glasses from two locations, (**b**). MgO contents in série 3.2-type glasses (**c**)–from the fourth to eight century western Mediterranean and Europe (blue), second to fifth century Balkans (yellow), and sixth century Balkans, in all 213 glasses from 18 different groups (green). Data sources: [1,2,3,4,5,6,7,8,9,10,14,17,19,23,25,26,28,29,30,33,34,47,48,49].

Changes in the proportion of glass types from the fourth to the sixth century in the central and eastern Balkans are depicted in Figure 8 (upper). The first change, from the fourth to the fifth century, is characterized by the disappearance of the Roman glass, an increase in the proportion of HIMT type, and the appearance of some Levantine type of glass in the record (12.2%). At the same time, série 3.2 remains an almost constant and dominating type throughout this period (changing from 50% to 51.2%).

Another change, from the fifth−sixth century, is characterized by the disappearance of HIMT and Levantine types and the appearance and almost total domination of a new type, série 2.1 (almost 90%), with the rest being série 3.2. It is plausible to explore if this change might be correlated with turbulent events of the fifth century in the Balkans, brought about by Hunnish plunder. The sixth century brought different kinds of change compared to the fifth. HIMT and Levantine types virtually disappeared, and a new type appeared that would dominate (85.7%). The older type, série 3.2, strongly diminished (9.2%). It would be plausible to explore if these changes happened gradually or abruptly.

The sixth century for northern Italy shows quite a different pattern from the contemporary central and eastern Balkans (Figure 8, middle). Contrary to the Balkans, with the strong domination of one type, there is a more balanced proportion of several types in Italy: Roman (37.9%), HIMT (29.9%), equal amounts of série 3.2 and série 2.1 (10.3%), and Levantine (5.7%). This might indicate that the Italian glass markets were more diversified than the contemporary Balkan ones, or that Balkan glass import was perhaps more centralized than in Italy. The apparent scarcity of the Levantine in the sixth century inner Balkans (2.6%) is notable and yet to be explained.

The continuous presence and the widespread findings of several major glass types during the fourth and the fifth centuries suggests that the Balkans imported glass artefacts and raw glass regularly from various Eastern Mediterranean production centers, implying regular economic activity. This further suggests that the traditional view of the dramatic economic decline during the late antiquity turmoil in peripheral parts of the Balkans might not be completely accurate. The analogous situation is described in the late antiquity Carthage after the Vandals conquest [49].

### 4.2. Evolving Chemical Composition of Série 3.2

**Figure 9:**
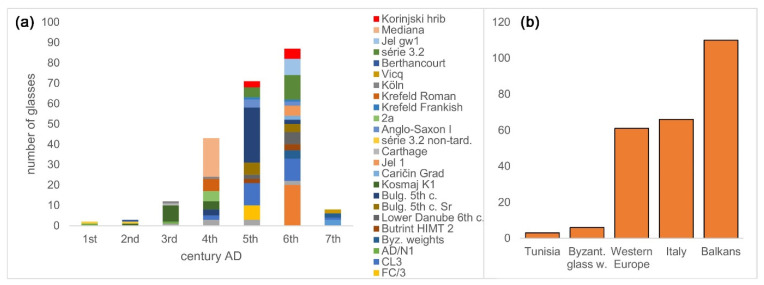
Distribution of série 3.2 in time and region. Histogram showing number of glasses belonging to the Foy série 3.2 against time, divided to particular groups (**a**). The absolute number of glasses of série 3.2 by the region (**b**). Data sources: [1,3,4,5,6,7,8,9,10,12,14,17,19,24,28,29,30].

More than 200 glasses of Foy 3.2 type that are now recognized (compared to the 19 originally described) give strong evidence to the hypothesis that série 3.2 was not limited to the turn of the fifth−sixth centuries and France and Tunisia [3]. It also weakens the arguments to include them under the umbrella name Foy 2 [10]. This type spread across the Mediterranean and beyond, to Germany, continental France, and Britain. It lasted from the second to the beginning of the seventh century, peaking during the fifth and the sixth centuries AD (Figure 9, left, right). It is distributed mostly around the Adriatic (Figure 9—middle), in Italy and the Balkans, highlighting the importance of the Adriatic trade route for the import of this type of glass and reflecting analogous findings regarding the distribution of Ca-rich HIMT, HIMT, and Foy 3.2 types [19,30]. This, together with the wide presence of Foy 3.2 glass across central parts of the Balkans, suggests that at least some Foy 3.2 raw glass was imported to the central Balkans over the Adriatic. However, during the sixth century, Foy 3.2 glasses in the Balkans showed a significant increase in the average MgO concentrations (1–1.25%) compared to the fifth century (Figure 8, lower), and to Foy 3.2 glasses outside the Balkans (0.6–0.7%), implying different sand quarries and, possibly, different trade routes for its import. This might reflect a marked shift in the Byzantine trade routes during the sixth century. The question of Foy 2.1 raw glass import is not clear. It could have been imported over the Adriatic, also over the Aegean and the Black Sea ports such as Odessus.

Foy 3.2 type was manufactured from the second century on, from geologically similar sand as Roman-Sb glass. Its production gradually increased through the period when manganese was replacing antimony as a decolorant and peaked during the fifth and the first half of the sixth century. Its production decreased rapidly during the second half of the sixth century, simultaneously to a rapid increase in Foy 2.1 production, and ceased altogether during the seventh century.

One explanation might be that this change took place when the search for new sand quarries finally led to the geologically different area, characterized by higher alumina, lime, iron, and magnesium. This hypothesis is built upon the observation that primary glass workshops were located in the vicinity of sand and wood resources, and that workshops regularly moved from place to place once resources were exhausted [31]. While it is well understood that the glass composition predominantly reflects the primary glassmaking source, rather than the secondary workshop, this model does not account for the probable and frequent local migration of workshops in search of raw materials. In Bet Eli’ezer (Hadera), 17 furnaces were in use for a year or two, and then moved to another location in the same area, in search of wood and sand [50]. It is thus reasonable to suppose that the primary glass composition would reflect these frequent location changes in somewhat increased compositional spread compared to a single workshop−single sand quarry production model. Therefore, as long as sands derived from the same geological process are exploited, this would result in the same primary glass type. The changes of glass composition in time reflecting such activities are termed compositional “evolution”. However, if at a particular moment in time, the quest for resources leads to exploiting sand with geologically markedly different characteristics (reflecting the different geological processes), we would all of a sudden have another basic type of primary glass, even from the same furnace. In such a case, a small geographical step might have led to a quite different type of primary glass.

To try to terminologically capture this complex picture of ever-varying exploitation conditions, we propose the term “generic composition/type” or “(geochemical) class” to glass manufactured from all quarries possessing a similar geological composition. Reflecting further the Linnaean approach, the term “family” denotes glass manufactured from all batches produced from the same sand quarry, and “species” denotes glass manufactured from a single batch.

### 4.3. Distribution of Fe-Rich 2.1

**Figure 10:**
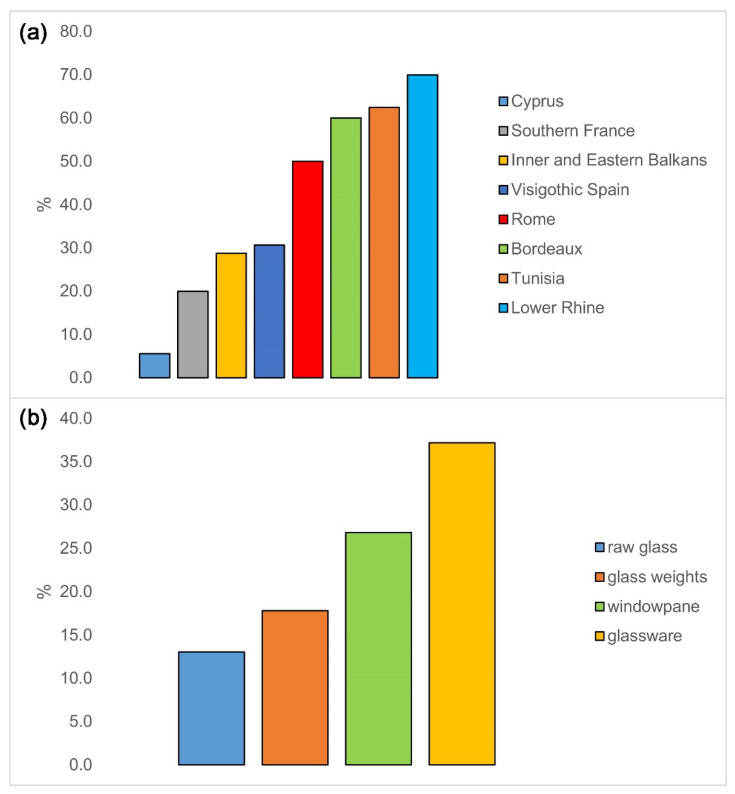
Percentages of Fe-rich glasses by region and type. Percentages of Fe-rich glasses among Foy 2.1 glasses by the region (**a**); percentages of Fe-rich glasses among Foy 2.1 glasses by object type (**b**). Data sources: [1,2,3,5,6,11,12,13,23,24,28].

Percentages of iron-rich glasses among Foy 2.1 type vary considerably from site to site, with a 25% overall average (Table 4). Taking into account only more numerous collections, the values span from 5.6% in Cyprus to 47.7% in Odartsi. Summing and comparing by regions, the values range from 5.6% in Cyprus to 70% in Lower Rhine (Figure 10, upper). Note, however, that only the Balkans and Visighotic Spain collections were more numerous, and in these two regions, the percentages were around 30%, which is close to the overall average of 25%. There is no notable correlation between the percentage of high-iron glass among Foy 2.1 type at some particular locations and its geographical distance from Egypt, where it was probably produced. This would indicate that this type of glass was freely exported and widely popular across the Mediterranean.

The percentages of high-iron samples are lower in the raw glass than in the manufactured glass (13% compared to 26.4%). This might suggest that iron was being added predominately, albeit not exclusively, in the secondary workshops, i.e., for color branding. Among the manufactured glass, the percentage is greater in glassware than in windowpane glass, perhaps also for marketing purposes (Figure 10, lower).

Glass products with elevated iron, color-branded or sand-derived, covered virtually the entire Mediterranean, from Cyprus, over the Balkans, Tunisia, Italy, Spain, France, and beyond, to inland France and Germany. The regional distribution might have been influenced by economic factors such as local purchasing power, as illustrated by the case of the dominance of more expensive Levantine glass in Carthage and Cyprus [10,32] compared to the inner Balkans. The production of this type of glass peaked during the sixth and seventh century AD, but lasted perhaps for some time before and after this period, showing the lasting popularity and competitiveness of this glass, apparently more economical in comparison to the more expansive and luxurious Levantine.

## Figures and Tables

**Table 1:** Ratios of oxides reflecting heavy and light sand minerals and the provenance indicator TiO_2_/Al_2_O_3_.

Group	Cent. AD	(Fe_2_O_3_ + TiO_2_ + MgO)/SiO_2_	(Al_2_O_3_ + K_2_O + CaO)/SiO_2_	Sum	TiO_2_/Al_2_O_3_
Roman glass n = 227	1st–4th	0.019	0.156	0.175	0.05
AD/N1 (Mn added) n = 45	1st–4th	0.017 ± 0.004	0.158 ± 0.014	0.174 ± 0.016	0.024 ± 0.009
AD/N2 (Sb added) n = 4	2nd–3rd	0.014 ± 0.003	0.112 ± 0.007	0.127 ± 0.010	0.047 ± 0.015
série 3.2 (non-t.) n = 2	1st–2nd	0.023 ± 0.003	0.124 ± 0.007	0.147 ± 0.009	0.045 ± 0.011
série 3.2 n = 17	5th/6th	0.021 ± 0.004	0.138 ± 0.016	0.159 ± 0.020	0.049 ± 0.009
série 2.1 n = 51	6th–7th	0.043 ± 0.011	0.173 ± 0.012	0.216 ± 0.016	0.062 ± 0.007

Foy série 3.2 is compared to two common Roman glass types, with added manganese (AD/N1), and with no added manganese (AD/N2), an assemblage of “Roman” glass, and with Foy group 2.1. Data sources: [3,14,18].

**Table 2:** Ratios and standard deviations of Nd/La, Ce/La, and Zr/TiO_2_ were sorted by increasing Nd/La values.

Original Classification	Group/Location	n	Nd/La ppm/ppm	Ce/La ppm/ppm	Zr/TiO_2_ ppm/pm
Foy 3.2	Group IV	2	0.81 ± 0.00	0.49 ± 0.02	56.88 ± 4.42
Foy 2.1 Fe-rich	Lower Danube	23	0.91 ± 0.03	1.44 ± 0.17	74.92 ± 4.78
Foy 3.2	Group I (MSG1c)	18	0.91 ± 0.03	1.77 ± 0.10	49.98 ± 6.58
Foy 2 High-Fe	Yeroskipou	2	0.92 ± 0.01	1.19 ± 0.22	52.58 ± 0.88
Foy 2.1	Lower Danube	43	0.92 ± 0.05	1.77 ± 0.12	74.71 ± 4.62
Ca-rich HIMT	Herdonia	4	0.93 ± 0.02	1.66 ± 0.10	72.80 ± 10.10
HIMT 2	San Giusto	9	0.93 ± 0.03	1.69 ± 0.09	60.90 ± 2.95
Foy 2 High-Fe	Byz. glass weights	31	0.93 ± 0.04	1.19 ± 0.09	55.86 ± 2.80
Roman Sb	AD/N2	4	0.93 ± 0.08	1.81 ± 0.04	63.70 ± 6.12
Foy 2	Yeroskipou	34	0.94 ± 0.02	1.68 ± 0.07	53.44 ± 3.57
Levantine	San Giusto	21	0.94 ± 0.05	1.74 ± 0.08	62.30 ± 5.86
Foy 2	Byz. glass weights	85	0.95 ± 0.04	1.63 ± 0.10	56.78 ± 3.69
HIT	Cyprus	2	0.96 ± 0.00	1.90 ± 0.01	53.96 ± 9.95
High Mn Lev 1	Cyprus	6	0.96 ± 0.02	1.71 ± 0.07	54.58 ± 4.54
Roman Mn	AD/N1	43	0.96 ± 0.06	1.82 ± 0.12	69.12 ± 67.43
HIMTb	Cyprus	5	0.98 ± 0.02	1.28 ± 0.08	45.95 ± 0.77
HIMTa	Cyprus	14	1.00 ± 0.05	1.77 ± 0.13	48.05 ± 4.29
Foy 3.2	AQ/3	1	1.25 ± 0.00	0.25 ± 0.00	52.67 ± 0.00
Foy 3.2	FC/3	4	1.30 ± 0.34	1.23 ± 0.70	58.19 ± 4.50
Foy 3.2	CL3	3	1.32 ± 0.48	1.40 ± 0.85	54.40 ± 3.11
Foy 3.2	Group III	2	1.33 ± 0.12	0.63 ± 0.53	52.08 ± 0.82
Foy 3.2	Group II	4	1.53 ± 0.17	1.79 ± 0.46	57.67 ± 4.25

Some Foy 3.2 glasses have similar values of Nd/La as Foy 2.1, while some have higher values. Data sources: [6,12,14,19,21,25,26,32,33,34].

**Table 3:** Foy 2.1 glass grouped according to iron concentrations.

	LI Série 2.1		HI		VHI		VHILL		Total Fe-Rich	
	n = 38		n = 47		n = 74		n = 4		n = 125	
wto%	aver.	std	aver.	std	aver.	std	aver.	std	aver.	std
Na_2_O	18.46	1.26	17.67	2.76	17.63	1.19	17.43	0.62	17.78	1.20
MgO	1.21	0.15	1.28	0.29	1.32	0.21	0.98	0.11	1.28	0.22
Al_2_O_3_	2.49	0.13	4.02	9.06	2.78	0.26	2.67	0.18	2.75	0.26
SiO_2_	64.50	1.08	63.81	9.55	64.61	1.22	67.88	0.79	64.93	1.48
K_2_O	0.76	0.14	1.00	0.87	0.91	0.17	0.66	0.03	0.89	0.17
CaO	7.97	0.65	7.34	1.21	7.48	0.59	5.43	0.20	7.42	0.68
TiO_2_	0.16	0.02	0.18	0.13	0.16	0.01	0.16	0.02	0.16	0.02
MnO	1.69	0.31	1.06	0.43	1.14	0.25	1.11	0.12	1.11	0.32
Fe_2_O_3_	1.03	0.11	1.64	0.21	2.69	0.41	2.57	0.15	2.29	0.61
ppm										
NiO			14	9	30	15	27	17	27	15
CuO	75	25	92	27	115	47	83	12	106	43
ZnO			60	110	48	18	46	14	46	16
SrO	798	86	701	147	715	99	433	222	705	120
ZrO_2_	111	14	108	24	117	18	100	67	114	20
SnO_2_			16	13	8	8	1	1	10	10
PbO	130	140	87	70	106	92	20	36	97	86
Sb_2_O_3_	188	289	155	75	134	119	12	13	135	107

Major and minor elements (wt%), and trace element concentrations (ppm) of Foy 2.1 glass. Groups: LI—low iron (Foy série 2.1 with Fe_2_O_3_ ≤ 1.3%); HI—high iron; VHI—very high iron; VHILL—very high iron low lime. Empty entries—data not available. Data sources: [1,2,3,5,6,12,13,23,24,28,32].

**Table 4:** Percentages of iron-rich among Foy 2.1 glasses, by archaeological site or collection.

Site/Collection	Type of Object	Fe-Rich Foy 2.1	Total Foy 2.1	Percent Fe-Rich
Jelica	windowpane, glassware	16	63	25
Caričin grad	raw glass	2	24	8
Serdica	glassware, raw glass	4	17	23
Odartsi	glassware	21	44	48
Dichin	glassware, windowpane	5	19	26
Cyprus	not specified	2	36	6
Krefeld-Gellep	glassware, bead	5	8	62
Köln	glassware	2	2	100
Crypta Balbi	glassware, windowpane	4	8	50
Bordeaux	raw glass	3	5	60
Marseille	glassware, raw glass, debris	1	6	17
Gémenos	glassware	4	4	100
Nabeul	glassware	4	6	67
Sidi Jdidi	glassware	1	2	50
Maguelone	glassware, raw glass, debris	1	20	5
Visigothic Spain	glassware	19	62	31
Byzantine glass weights	glass weights	31	174	18
Total		125	500	25.0

Data sources: [1,2,3,5,6,11,12,13,23,24,28].

## Supplementary Materials

The following supporting information can be downloaded at: www.mdpi.com/article/10.3390/ma15031086/s1; Table S1: Foy 3.2 recycling.

## References

[1] Balvanović R., Stojanović M.M., Šmit Ž. (2018). Exploring the unknown Balkans: Early Byzantine glass from Jelica Mt in Serbia and its contemporary neighbours. J. Radioanal. Nucl. Chem..

[2] Balvanović R., Šmit Ž. (2020). Sixth-century AD glassware from Jelica, Serbia—an increasingly complex picture of late antiquity glass composition. Archaeol. Anthr. Sci..

[3] Foy D., Picon M., Vichy M., Thirion-Merle V., Montagnac (2003). Caractérisation des verres de la fin de l’Antiquité en Méditerranée occidentale: L’émergence de nouveaux courants commerciaux. Échanges et Commerce du Verre Dans le Monde Antique: Actes du Colloque International de l’Association Française pour l’Archéologie du Verre.

[4] Stojanović M.M., Šmit Ž., Glumac M., Mutić J. (2015). PIXE–PIGE investigation of Roman Imperial vessels and window glass from Mt. Kosmaj, Serbia (Moesia Superior). J. Archaeol. Sci. Rep..

[5] Drauschke J., Greiff S., Zorn B., Hilgner A. (2010). Early Byzantine glass from Caričin Grad/Iustiniana Prima (Serbia): First results concerning the composition of raw glass chunks. Glass along the Silk Road from 2000 BC to AD 1000.

[6] Cholakova A., Rehren T., Freestone I.C. (2016). Compositional identification of 6th c AD glass from the Lower Danube. J. Archaeol. Sci. Rep..

[7] Cholakova A., Rehren T., Rosenow D., Phelps M., Meek A., Freestone I. (2018). A Late Antiquity manganese-decolourised glass composition: Interpreting patterns and mechanisms of distribution. Things that Travelled: Mediterranean Glass in the First Millennium ce.

[8] Conte S., Chinni T.R., Arletti R., Vandini M. (2014). Butrint (Albania) between eastern and western Mediterranean glass production: EMPA and LA-ICP-MS of late antique and early medieval finds. J. Archaeol. Sci..

[9] Milavec T., Šmit Ž. (2020). Analyses of glass from late antique hilltop site Korinjski hrib above Veliki Korinj (Slovenia). Arheol. Vestn..

[10] Schibille N., Sterrett-Krause A., Freestone I.C. (2016). Glass groups, glass supply and recycling in late Roman Carthage. Archeol. Antrhropol. Sci..

[11] Ceglia A., Cosyns P., Schibille N., Meulebroeck W. (2017). Unravelling provenance and recycling of late antique glass from Cyprus with trace elements. Archaeol. Anthropol. Sci..

[12] Schibille N., Meek A., Tobias B., Entwistle C., Avisseau-Broustet M., Da Mota H., Gratuze B. (2016). Comprehensive chemical characterisation of Byzantine glass weights. PLoS ONE.

[13] Ares J.J., Guirado V.E., Gutiérrez Y.C., Schibille N. (2019). Changes in the supply of eastern Mediterranean glasses to Visigothic Spain. J. Archaeol. Sci..

[14] Gallo F., Silvestri A., Molin G. (2013). Glass from the Archaeological Museum of Adria (North-East Italy): New insights into Early Roman production technologies. J. Archaeol. Sci..

[15] Silvestri A. (2008). The coloured glass of Iulia Felix. J. Archeol. Sci..

[16] Silvestri A., Molin G., Salviulo G. (2008). The colourless glass of Iulia Felix. J. Archeol. Sci..

[17] Jackson C.M. (2005). Making colourless glass in the Roman period. Archaeometry.

[18] Nenna M.D., Vichy M., Picon M. (1997). L’atelier de verrier de Lyon du 1er siècle après J.-C, et l’origine des verre “romains”. Rev. d’Archaéométrie.

[19] Maltoni S., Chinni T., Vandini M., Cirelli E., Silvestri A., Molin G. (2015). Archaeological and archaeometric study of the glass finds from the ancient harbour of Classe (Ravenna- Italy): New evidence. Herit. Sci..

[20] Rehren T., Brüggler M. (2020). The Late Antique glass furnaces in the Hambach Forest were working glass-not making it. J. Archaeol. Sci. Rep..

[21] Gallo F., Marcante A., Silvestri A., Molin G. (2014). The glass of the “Casa delle Bestie Ferite”: A first systematic archaeometric study on Late Roman vessels from Aquileia. J. Archaeol. Sci..

[22] Barfod G.H., Freestone I.C., Lesher C.E., Lichtenberger A., Raja R. (2020). ‘Alexandrian’ glass confirmed by hafnium isotopes. Sci. Rep..

[23] Mirti P., Lepora A., Saguì L. (2000). Scientific analysis of fragments from the seventh-century glass Crypta Balbi in Rome. Archaeometry.

[24] Velde B. (1990). Aluminum and calcium oxide content of glass found in western and northern Europe, first to ninth centuries. Oxf. J. Archaeol..

[25] Silvestri A., Tonietto S., Molin G. (2011). The palaeo-Christian glass mosaic of St. Prosdocimus (Padova, Italy): Archaeometric characterization of ‘gold’ tesserae. J. Archaeol. Sci..

[26] Maltoni S., Silvestri A., Marcante A., Molin G. (2016). The transition from Roman to Late Antiquity glass: New insights from the *Domus of Tito Macro* in Aquilea (Italy). J. Archaeol. Sci..

[27] Foster H.E., Jackson C.M. (2010). The composition of late Romano-British colourless vessel glass: Glass production and consumption. J. Archaeol. Sci..

[28] Wedepohl K.H., Pirling R., Hartmann G. (1997). Römische und fränkische Glaäser aus dem Gräberfeld von Krefeld-Gellep. Bonn. Jahrbücher.

[29] Stamenković S.Z. (2015). Glass Production Technology and Production Centres in Dacia Mediterranea. Ph.D. Thesis.

[30] Freestone I.C., Hughes M.J., Stapleton C.P., Evison V.I. (2008). The composition and production of Anglo-Saxon glass. Catalogue of Anglo-Saxon Glass in the British Museum.

[31] Gorin-Rosen Y., Nenna M.-D. (2000). The Ancient Glass Industry in Israel: Summary of the Finds and New Discoveries. La Route du Verre: Ateliers Primaires et Secondaires du Second Millénaire av. J.-C. au Moyen Âge.

[32] Ceglia A., Cosyns P., Nys K., Terryn H., Thienpont H., Meulebroeck W. (2015). Late antique glass distribution and consumption in Cyprus: A chemical Study. J. Archaeol. Sci..

[33] Gliozzo E., Turchiano M., Giannetti F., Santagostino B.A. (2015). Late Antique glass vessels and production indicators from the town of Herdonia (Foggia, Italy): New data on CaO-rich/weak HIMT glass. Archaeometry.

[34] Gliozzo E., Braschi E., Giannetti F., Langone A., Turchiano M. (2019). New geochemical and isotopic insights into the Late Antique Apulian glass and the HIMT1 and HIMT2 glass productions—The glass vessels from San Giusto (Foggia, Italy) and the diagrams for provenance studies. Archaeol. Anthropol. Sci..

[35] Brill R.H., Weinberg G.D. (1988). Scientific investigations of Jalame glass and related finds. Excavations in Jalame: Site of a Glass Factory in Late Roman Palestine.

[36] Baxter M.J., Buck C.E., Ciliberto E., Spoto G. (2000). Data handling and statistical analysis. Modern Analytical Methods in Art and Archaeology.

[37] Foster H., Jackson C. (2009). The composition of ‘naturally coloured’ late Roman vessel glass from Britain and the implications for models of glass production and supply. J. Archeol. Sci..

[38] Rehren T., Cholakova A. (2010). The early Byzantine HIMT glass from Dichin, Northern Bulgaria, UCL. Interdiscip. Stud..

[39] Shortland A., Rogers N., Eremin K. (2007). Trace element discriminants between Egyptian and Mesopotamian Late Bronze Age glasses. J. Archaeol. Sci..

[40] Åström M. (2001). Abundance and fractionation patterns of rare earth elements in streams affected by acid sulphate soils. Chem. Geol..

[41] Åström M., Corin N. (2003). Distribution of rare earth elements in anionic, cationic and particulate fractions in boreal humus-rich streams affected by acid sulphate soils. Water Res..

[42] Wedepohl K.H., Baumann A. (2000). The use of marine molluskan shells for Roman glass and local raw glass production in the Eifel area (western Germany). Naturwissenschaften.

[43] Wedepohl K.H., Gaitzsch W., Follmann-Shultz A.-B. (2003). Glassmaking and Glassworking in Six Roman Factories in the Hambach Forest, Germany. Ann. AIHV.

[44] Schibille N., Freestone I.C. (2013). Composition, production and procurement of glass at San Vincenzo Al Volturno: An early Medieval monastic complex in Southern Italy. PLoS ONE.

[45] Freestone I.C., Degryse P., Lankton J., Gratuze B., Schneider J., Rosenow D., Phelps M., Meek A., Freestone I. (2018). HIMT, glass composition and the commodity branding in the primary glass industry. Things That Travelled, Mediterranean Glass in the First Millennium CE.

[46] Kamber B.S., Greig A., Collerson K.D. (2005). A new estimate for the composition of weathered young upper continental crust from alluvial sediments, Queensland, Australia. Geochim. Cosmochim. Acta.

[47] Arletti R., Vezzalini G., Benati S., Mazzeo Saracino L., Gamberini A. (2010). Roman Window Glass: A Comparison of Findings from Three Different Italian Sites. Archaeometry.

[48] Smith T., Henderson J., Faber E.W., Gan F., Li Q., Henderson J. (2016). Early Byzantine glass supply and consumption: The case of Dichin, Bulgaria. Recent Advances in the Scientific Research On Ancient Glass And Glaze.

[49] Siu I., Henderson J., Faber E. (2017). The production and circulation of Carthaginian glass under the rule of the romans and the vandals (fourth to sixth century ad): A chemical investigation. Archaeometry.

[50] Freestone I.C. Primary glass sources in the mid first millennium AD. Proceedings of the Annales du 15e Congrès de l’Association Internationale pour l’Histoire du Verre.

